# Beneficial effects of proanthocyanidins on skin aging: a review

**DOI:** 10.3389/fnut.2025.1650328

**Published:** 2025-09-03

**Authors:** Hua Ye, Jiaqiang Sun, Linlin He, Chenyue Ai, Wengang Jin, A. M. Abd El-Aty

**Affiliations:** ^1^College of Biological Science and Engineering, Shaanxi University of Technology, Hanzhong, China; ^2^Shaanxi Province Key Laboratory of Bio-Resources, Shaanxi University of Technology, Hanzhong, China; ^3^Qinba Mountain Area Collaborative Innovation Center of Bioresources Comprehensive Development, Shaanxi University of Technology, Hanzhong, China; ^4^Qinba State Key Laboratory of Biological Resources and Ecological Environment (Incubation), Shaanxi University of Technology, Hanzhong, China; ^5^Department of Pharmacology, Faculty of Veterinary Medicine, Cairo University, Giza, Egypt; ^6^Department of Medical Pharmacology, Faculty of Medicine, Atatürk University, Erzurum, Türkiye

**Keywords:** proanthocyanidins (PCs), oxidative stress, collagen and elastin, inflammatory response, skin pigmentation

## Abstract

Skin aging is a gradual physiological process influenced by both intrinsic and environmental factors and is characterized by the appearance of wrinkles, pigmentation, reduced elasticity, dryness, and vascular changes. In recent years, interest in the use of natural bioactive compounds to mitigate skin aging has increased, reflecting the global shift toward safer, sustainable, and health-conscious skincare solutions. Proanthocyanidins (PCs), a class of polyphenolic compounds derived from plant sources, exhibit strong antioxidant, anti-inflammatory, and antipigmentation properties. These compounds have considerable potential for enhancing the structure and function of aged skin by reducing oxidative stress, promoting collagen and elastin synthesis, alleviating the skin inflammatory response, and inhibiting pigmentation. Despite their promising therapeutic value, the efficacy of PCs can be compromised by their environmental instability and low bioavailability. Advances in encapsulation techniques and combination formulations have shown promise in enhancing the stability and delivery efficiency of PCs, thereby improving their performance in antiaging applications. In conclusion, PCs offer a scientifically grounded and sustainable approach for addressing skin aging. Their integration into dermatological products represents an innovative and eco-conscious strategy for developing next-generation skincare solutions with broad health and consumer benefits.

## 1 Introduction

Skin aging is a complex phenomenon resulting from the interplay of internal (genetic) and external (environmental) factors. It can be categorized into two distinct types: endogenous and exogenous skin aging (1, 2). Endogenous skin aging is influenced by genetic factors and progresses gradually with advancing age. Its characteristic manifestations include a decline in overall skin texture, the appearance of fine and evenly distributed wrinkles, a slightly dull complexion, and an absence of prominent pigmentation spots or erythema. In contrast, exogenous skin aging, on the other hand, is associated with detrimental external environmental factors and poor lifestyle habits, such as ultraviolet (UV) radiation exposure, air pollution, sleep deprivation, and smoking. Among these factors, UV radiation stands out as the most significant contributor to this form of aging; it is commonly referred to as photoaging of the skin. The typical manifestations of photoaged skin include a leather-like appearance in areas exposed to UV light, characterized by dense localized wrinkles along with common pigmentation spots and erythema (3, 4). UVA (320–400 nm) possesses substantial penetration capability, allowing it to directly reach the dermis and induce the production of reactive oxygen species (ROS) (5). Conversely, UVB (275–320 nm) primarily accumulates in the epidermis and is recognized as the principal pathogenic factor responsible for sunburn erythema, hence referred to as the “erythema effect” of UV radiation (6). In contrast to UVA and UVB rays, most UVC (230–275 nm) wavelengths are absorbed by the surface of the ozone layer before it reaches the Earth’s surface (7).

Compared with endogenous aging, skin aging induced by UV exposure is substantially more severe. Endogenous aging typically involves progressive degeneration characterized by gradual degradation of the extracellular matrix without causing pronounced visible damage. Conversely, photoaging resulting from prolonged UV exposure leads to extensive collagen degradation and the denaturation of elastin, which promotes acute damage to the extracellular matrix and results in substantial harm. Furthermore, UV radiation induces excessive ROS within skin tissues, facilitating local capillary dilation and triggering an inflammatory response that manifests as redness, swelling, and burning sensations on the skin (8). Additionally, UV radiation can increase melanocyte activity, leading to excessive melanin production and accumulation. This process results in pigmentation changes on sun-exposed areas of the skin, giving rise to pigmented spots (9, 10). Skin aging profoundly impacts individuals’ appearance and causes significant physical and psychological distress. Consequently, research focused on preventing and treating skin aging has garnered considerable attention from scientific researchers (11).

Currently, in addition to maintaining a healthy lifestyle, cultivating a positive mindset, and utilizing skincare products tailored to individual needs, the prevention and treatment methods for natural skin aging primarily encompass medical aesthetic interventions along with the administration of metformin (12, 13) and doxycycline (DOX) (14). The strategies for preventing and treating skin photoaging mainly involve sun protection measures alongside topical medications such as retinoic acid (15), chloroacetic acid (16), and 5-fluorouracil (17). Moreover, medical aesthetic techniques are employed within these treatments. While medical aesthetic approaches yield rapid results in the short term, they also carry certain risks. These risks include but are not limited to local skin infections at injection sites, induration, ecchymosis, vascular embolism, nerve damage, fat embolism, allergic reactions, and other complications—potentially resulting in unnatural facial expressions. Adverse reactions associated with pharmacological treatments for skin aging can be severe and may present pronounced side effects. Consequently, it is essential to explore new anti-skin aging products.

Compared with traditional pharmaceuticals used to combat skin aging, natural bioactive substances have emerged as promising alternatives because of their reduced side effects and diverse biological activities (18, 19). Natural bioactive compounds such as retinoids, peptides and antioxidants (such as vitamin C) and PCs play beneficial roles in the prevention and treatment of skin aging. Retinoids promote keratin renewal, stimulate collagen production, and inhibit its degradation (20, 21). Peptides serve as antiaging agents primarily by increasing the levels of collagen, elastin, and hyaluronic acid, thereby enhancing the elasticity and firmness of the skin (22). Vitamin C is a well-known antioxidant that effectively neutralizes free radicals, mitigates oxidative stress damage to cells, and acts as an essential cofactor in collagen synthesis. It promotes collagen production while inhibiting melanin formation, thus reducing pigmentation and increasing the degree of complexion (23). However, all the aforementioned drugs or ingredients have varying degrees of side effects, which can not only impact therapeutic efficacy but also lead to adverse reactions within the human body. Retinoids are known for their high irritability to the skin, which may result in adverse reactions such as dryness and peeling (20, 24). The effectiveness of peptides tends to be relatively slow; furthermore, they require specific concentrations and careful formulation design for optimal results (22). Vitamin C is prone to oxidation, which can diminish its efficacy. Additionally, high concentrations of vitamin C may cause irritation both on the skin surface and in the gastric mucosa (23, 25).

PCs are internationally recognized as effective natural polyphenolic antioxidants with notable properties. In comparison with other antioxidants, PCs are widely available from various sources at low cost while maintaining a relatively high safety profile—making them suitable for long-term consumption or application (26). PCs possess a robust capacity to neutralize free radicals effectively while inhibiting ROS formation (27). Compared with retinoids, peptides, and vitamin C, PCs exhibit unique antiaging potential that manifests not only through the enhancement of glycolipid metabolism and microvascular perfusion—achieved by inhibiting adipogenesis and improving mitochondrial function, respectively (28, 29)—but also by mitigating oxidative stress-induced damage to skin cells via the elimination of free radicals. This protective action safeguards both the structure and function of skin cells. Furthermore, PCs can inhibit the release of inflammatory factors, thereby alleviating the inflammatory response in the skin and consequently slowing its aging process (30, 31).

**Figure 1:**
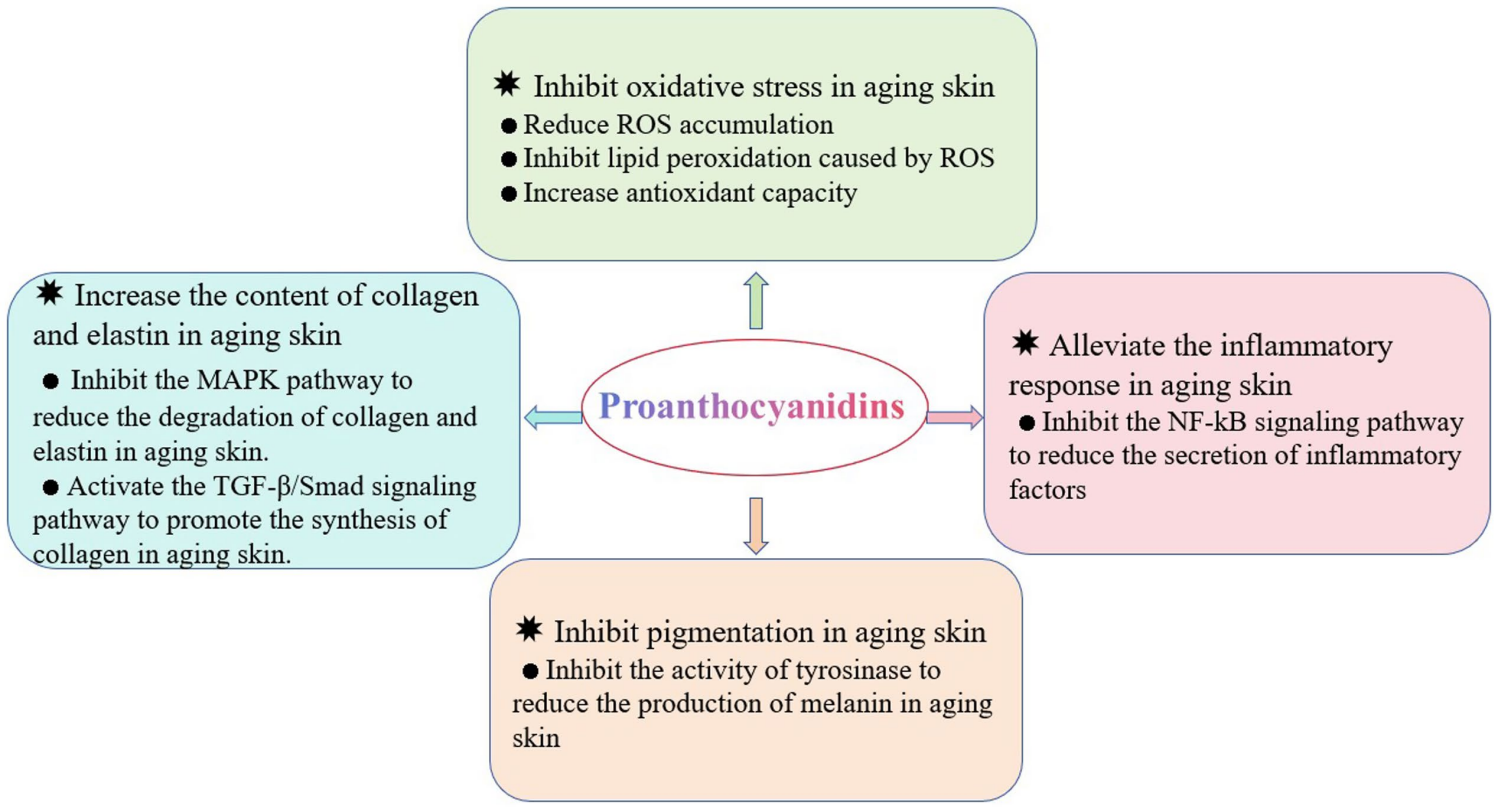
The beneficial effects of PCs on the repair of aging skin. Four main effects include inhibiting oxidative stress, increasing collagen and elastin content, alleviating the inflammation, and inhibiting pigmentation. ROS, reactive oxygen species; MAPK, mitogen-activated protein kinase; NF-κB, nuclear transcription factor-κB; TGF-β, transforming growth factor-β.

The reparative effects of PCs on aging skin have garnered significant attention in recent years. It exerts beneficial effects through various mechanisms: inhibiting the oxidative stress associated with aging skin; enhancing the collagen and elastin contents; alleviating inflammatory responses; and reducing the prevalence of pigmentation issues in aged skin (Figure 1). This paper reviews the structure and stability of PCs as well as their underlying mechanisms contributing to their ability to repair aging skin. Furthermore, strategies aimed at enhancing the stability and bioavailability of these products are discussed, providing novel insights for research focused on utilizing PCs to combat skin aging.

## 2 Resources, structure and stability of PCs

Fruits and seeds containing PCs include berries (e.g., grapes, blueberries, blackberries, cranberries), nuts (e.g., almonds, hazelnuts), as well as apples, hawthorns, cocoa beans, black beans, red beans, black-skinned peanuts, and red-skinned peanuts. Grape seeds are indeed one of the richest natural sources of PCs among known plant species, with a content exceeding 95% on the basis of dry weight, which is significantly greater than that found in other commonly known plants. PCs have been isolated and purified from raw materials via techniques such as supercritical CO₂ extraction, solvent extraction, column chromatography, high-speed countercurrent chromatography, membrane separation, and crystallization or recrystallization.

**Figure 2:**
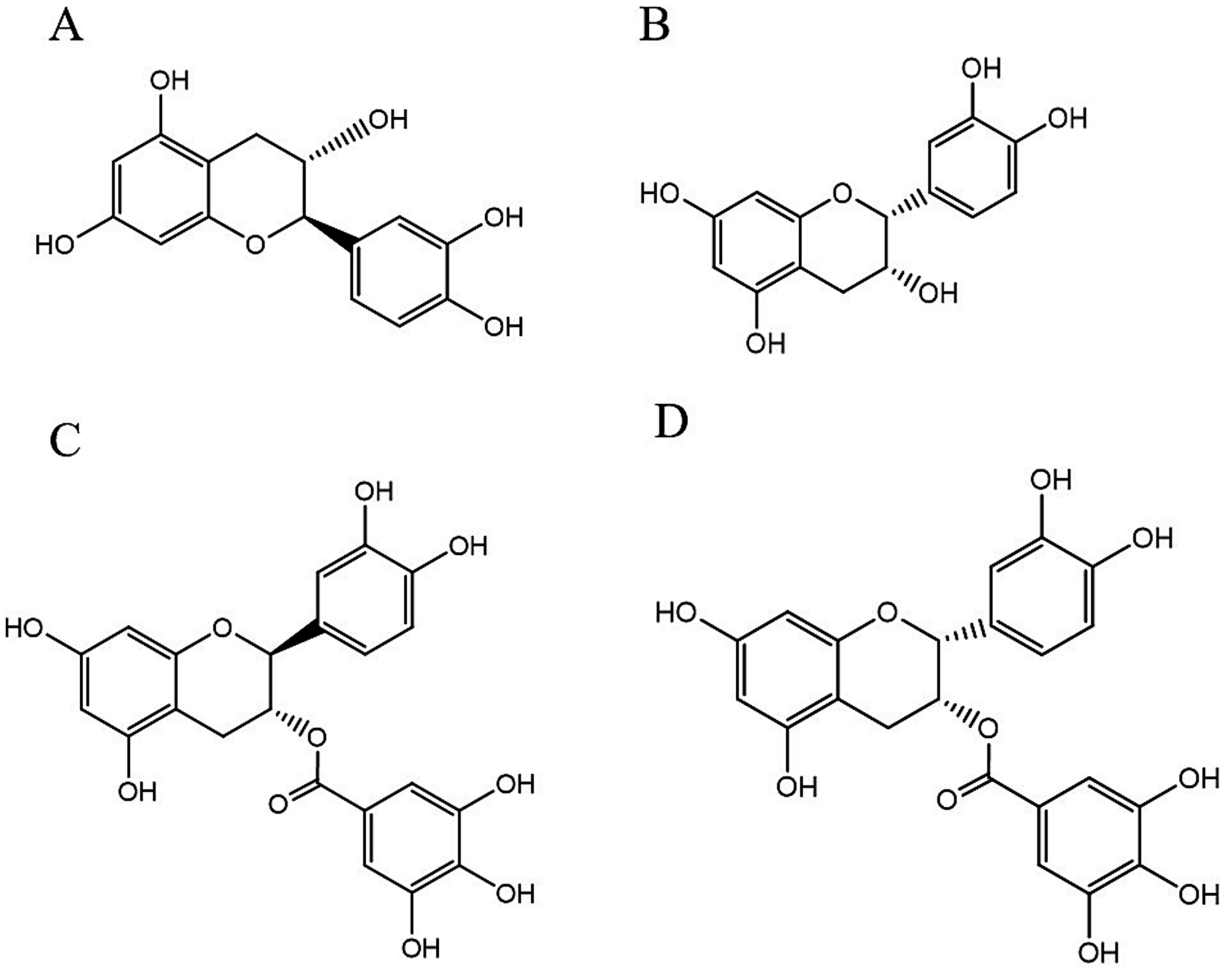
The structural formulas of different types of monomers that make up PCs. Each structure is a flavonoid compound, with variations in hydroxyl group arrangements and benzene rings. The compounds display different stereochemical configurations, indicated by solid and dashed wedges. **(A)** (+) -catechin (C), **(B)** (−) -epicatechin (EC), **(C)** (+) -catechin gallic acid (CG), **(D)** (−) -epicatechin gallic acid (ECG).

PCs, commonly known as condensed tannins, are polymers characterized by both low and high molecular weights. It is formed through the linkage of multiple hydroxylated flavane-3-alcohol units via carbon–carbon bonds. The monomers that constitute these polymers encompass four distinct structural types: (+)-catechin (designated C), (−)-epicatechin (designated EC), (+)-catechin gallate (designated CG), and (−)-epicatechin gallate (designated ECG) (32), as illustrated in Figure 2. The simplest form of PCs consists of catechin (C) or epicatechin (EC) monomers, which can undergo polymerization to yield dimers, trimers, tetramers, and so forth, extending up to decamers. PCs within the range of 2 °C to 4 °C are typically classified as oligomeric PCs on the basis of the degree of polymerization; those above 4 °C are categorized as polymeric PCs (33). PCs are further divided into two categories: Type A and Type B. Type A PCs comprise trimers (such as PCs A1--A2) that contain double bonds—specifically, a C=C double bond and a C=O double bond—as illustrated in Table 1. This type is found in select plants such as peanuts and lychees (34–36). Currently, the structures of eight type B dimers have been successfully isolated and elucidated; they are designated B1 through B8 (37), as presented in Table 1. The primary structural distinction among these dimers lies in their varying carbon connection sites. Dimers B1 to B4 are linked via the C4--C8 site, whereas dimers B5 to B8 connect through the C4--C6 site (38).

**Table 1:** Different structures of PCs.

Compound	Source	Structure		Ref.
Proanthocyanidin A1	Peanut skin, lychee seeds	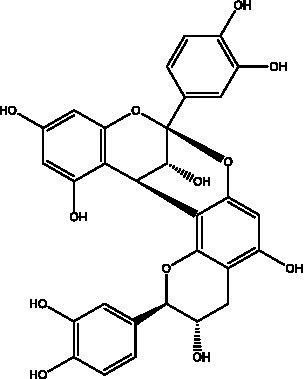		(124, 125)
Proanthocyanidin A2		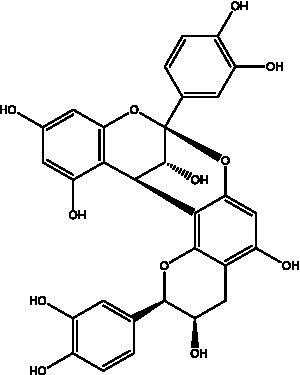		
Proanthocyanidin B1	Flat peaches, black soybean seed coats, strawberries, *Petiveria alliacea*, hazelnut skin, soybean seed coats	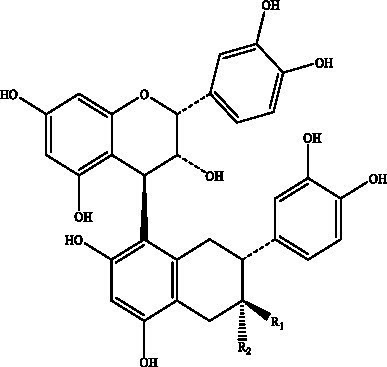	R_1_ = OH R_2_ = H	(126–131)
Proanthocyanidin B2			R1 = H R2 = OH	
Proanthocyanidin B3		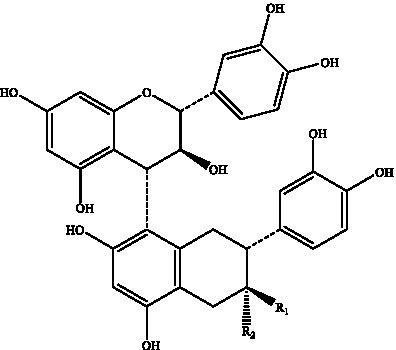	R_1_ = OH R_2_ = H	
Proanthocyanidin B4			R1 = H R2 = OH	
Proanthocyanidin B5		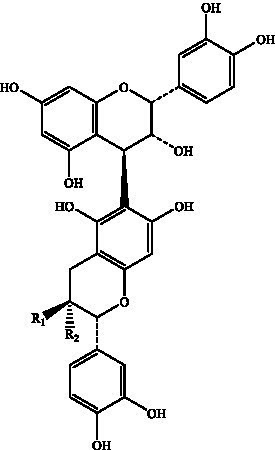	R_1_ = OH R_2_ = H	
Proanthocyanidin B6			R_1_ = H R_2_ = OH	
Proanthocyanidin B7		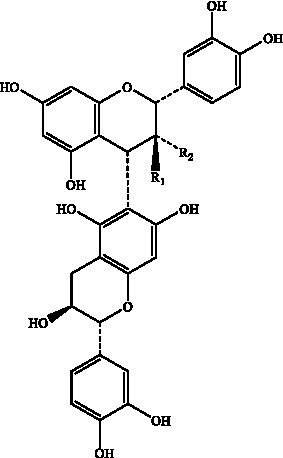	R_1_ = OH R_2_ = H	
Proanthocyanidin B8			R_1_ = H R_2_ = OH	

PCs are recognized for their instability, and environmental factors such as pH, temperature, and light play a significant role in influencing their stability (39). The stability of cocoa bean PCs is optimal within a neutral to weakly acidic environment. In contrast, under alkaline conditions, the B-ring hydroxyl group of PCs becomes vulnerable to oxidation, resulting in a decrease in their antioxidant activity (40). In experiments involving purple sweet potato PCs treated with citric acid–phosphate buffer solutions at various pH values (3.0, 5.0, and 7.0), at pH 3.0, purple sweet potato PCs exhibited relative stability characterized by a slow degradation rate. However, at pH 7.0, the phenolic hydroxyl group (-OH) within the PCs molecule undergoes oxidation to form a quinone structure; this transformation diminishes both its thermal stability and reactivity (41). Elevated temperatures further exacerbate the degradation of PCs (42). For example, when PCs purified from black carrots, elderberries, and strawberries were subjected to heating at 95 °C for 6 hours at pH 3.5, there was an observable lightening of sample color along with the formation of degradation products such as chalketonoside, phenolic acids, and maroumarin. Although these newly formed degradation products exhibited some antioxidant capacity, they were insufficient to compensate for the loss of activity associated with PCs (43).

The drying process of blueberry pulp containing PCs at various temperatures (175 °C, 200 °C, and 225 °C) leads to continuous degradation of the compound. Notably, the rate of degradation increases with increasing temperature, whereas the antioxidant activity correspondingly decreases with both elevated temperature and prolonged exposure time. These findings indicate that high-temperature dry heat treatment adversely affects the stability of PCs and consequently reduces their bioavailability (44). When the storage temperature exceeds 35 °C, the degradation rate of PCs in blueberries significantly increases, indicating that elevated temperatures can compromise the stability of PCs in these fruits. Therefore, low-temperature storage is essential for preserving the PCs content (45). Additionally, during storage and transportation, PCs are inevitably subjected to degradation caused by light exposure. When the duration of exposure exceeds 96 h, a marked increase in the degradation rate of PCs occurs (46). Consequently, to maintain the activity of PCs, it is imperative to sustain an acidic environment throughout processing, storage, and transportation; maintain temperatures below 65 °C; and minimize light exposure (47).

In addition, processing technology significantly impacts the stability of PCs in food (39). Research has shown that the PCs content in processed blackcurrant products, such as juice and jam, can decrease by more than 90% compared with that in fresh fruits. This finding indicates that traditional processing methods for juice and jam may lead to a substantial loss of PCs from blackcurrants (48). Cocoa powder is recognized for its high PCs content. During the fermentation process of cocoa powder, hydrolases secreted by microorganisms—such as lactic acid bacteria and yeast—including glycosidases and esterases—can cleave the glycosidic or ester bonds within PCs. This degradation results in the formation of small-molecule phenolic acids or oligomers. Furthermore, oxidases produced by these microorganisms, such as polyphenol oxidase, may catalyze the oxidation of PCs into quinone compounds. Consequently, this process reduces its antioxidant activity and affects its bioaccessibility (49). Extrusion treatment has been shown to significantly increase the levels of PCs monomers and dimers present in grape seeds and skins. However, it simultaneously reduces the total PCs content in grape skins and residues by 18 to 53%. These findings demonstrate that while food processing can release PCs by disrupting the food matrix, it may also induce alterations in their chemical structure, which could compromise their stability and subsequently affect their bioavailability (50).

## 3 Repair mechanism of PCs in skin aging

Natural skin aging is a physiological decline characterized by the deterioration of bodily functions and metabolism, which is largely irreversible. In contrast, photoaging refers to pathological damage induced by UV radiation. Certain symptoms associated with photoaging can be alleviated through pharmacological interventions and stringent sun protection measures aimed at repairing photodamage. Currently, pharmacological treatments focus primarily on addressing skin photoaging resulting from UV exposure. However, relatively few studies have investigated skin aging caused by other factors. Research has demonstrated that PCs treatments can inhibit oxidative stress in aging skin, increase the collagen and elastin contents within aged dermal layers, mitigate inflammatory responses related to skin aging, and suppress pigmentation changes in aged skin. Collectively, these actions contribute to the reparative effect of PCs in aging skin.

### 3.1 PCs inhibit oxidative stress in aging skin

Excessive accumulation of ROS in the body is a primary factor contributing to oxidative stress in the skin (51). ROS are a group of highly reactive molecules containing oxygen, including superoxide radicals (O2-), hydroxyl radicals (OḤ), and hydrogen peroxide (H₂O₂) (52). In naturally aging skin, ROS predominantly originate from mitochondria, while intracellular enzyme systems also generate ROS during catalytic reactions (53). In photoaged skin, UV radiation interacts with oxygen molecules within the body, leading to the generation and accumulation of ROS (54). The presence of ROS can initiate lipid peroxidation, which represents one of the critical mechanisms underlying cell membrane damage and is closely associated with cellular senescence (55).

As bioactive substances, the prominent characteristic of PCs is their capacity to neutralize ROS and mitigate the production of oxidative stress products, thereby decelerating the process of skin aging. In experiments involving H₂O₂-treated human skin fibroblasts, treatment with sea buckthorn PCs significantly decreased ROS levels and notably restored the morphology of senescent cells while enhancing cellular activity. This intervention effectively alleviated cellular senescence and delayed the progression of skin aging (56). Li et al. (57) induced senescence in rat adrenal pheochromocytoma cell lines and mouse embryonic fibroblast lines via the use of rapamycin and etoposide, followed by treatment with peanut skin PCs. The findings revealed that peanut skin PCs decreased the ROS levels in these two cell lines. Furthermore, it diminished the proportion of senescent cells while restoring their proliferative capacity, thus effectively mitigating cellular senescence. Additionally, PCs have been shown to inhibit lipid peroxidation caused by ROS, further suppressing skin aging. Anshu Mittal et al. (58) investigated the effects of grape seed PCs on photoaging in hairless SKH-1 mice exposed to UVB radiation. The results indicated that dietary supplementation with 0.2 and 0.5% grape seed PCs significantly inhibited the formation of lipid peroxides induced by UVB exposure and reduced ROS levels in a dose-dependent manner in these murine models. These findings suggest that grape seed PCs may delay the aging process of skin cells by decreasing the generation of lipid peroxidation products such as malondialdehyde (MDA), which are associated with UVB-induced ROS. Li et al. (59) investigated the effects of red millet PCs on aging model mice subjected to UVB radiation. The results demonstrated that PCs significantly enhanced the activity of antioxidant enzymes in these aging model mice while reducing MDA levels, thereby highlighting their potential in combating skin photoaging. Sharma et al. (60) induced photoaging in SKH-1 hairless mouse skin through UVB exposure and subsequently administered diets containing 0.2 and 0.5% (w/w) grape seed PCs. The results revealed a significant reduction in ROS, H₂O₂, and MDA levels within the mouse skin, suggesting that grape seed PCs could effectively increase the antioxidant capacity of photoaged skin and delay the aging process.

PCs can also stimulate key antioxidant enzymes, including superoxide dismutase (SOD) and glutathione peroxidase (GSH-Px). The synthesis of GSH-Px and glutathione reductase (GSH-Rd), along with the increase in their activities, contributes to a reduction in MDA levels. This process ultimately increases the antioxidant capacity of the skin and plays a significant role in combating skin aging. Experimental results from Hwang et al. (61) evaluated the antioxidant effects of proanthocyanidin oligomers on human retinal pigment epithelial cells and demonstrated that these oligomers significantly increased SOD and GSH-Px activities within the cells, indicating their substantial antioxidant activity. Li et al. (62) established a natural aging model in mice through a high-fat diet and subsequently treated them with PCs (200 mg/kg in the diet) extracted from lotus seed skins. Their findings revealed that PCs markedly increased both GSH-Px and SOD activities in the mouse liver while simultaneously reducing MDA levels. These findings suggest that PCs derived from lotus seed skins possess strong antioxidant properties that are capable of delaying skin aging. Chen et al. (63) investigated the effects of lotus seed PCs on UV radiation-induced damage in human skin fibroblast epithelial cells. They reported that treatment with lotus seed PCs led to a reduction in the amount of ROS generated by UV exposure, an increase in the intracellular SOD level, and decreased MDA production, thereby mitigating oxidative stress-related damage to the skin. Shi et al. (64) investigated the effects of persimmon PCs on *in vitro* injury to human keratinocyte HaCaT cells induced by UVB radiation. These findings demonstrated that persimmon PCs significantly elevated the levels of antioxidant enzymes, including SOD and GSH-Px, while concurrently inhibiting the production of ROS and MDA. Consequently, this treatment effectively alleviated the oxidative stress response in these cells. Additionally, in SKH hairless mice exhibiting skin photoaging due to UVB radiation, dietary inclusion of 0.2 and 0.5% grape seed PCs was found to increase the levels of antioxidant enzymes such as GSH-Px. These findings suggest that grape seed PCs can substantially reduce oxidative stress resulting from UVB exposure while providing protective effects against skin damage caused by UVB radiation (58). Furthermore, a clinical trial involving 39 healthy adults who consumed roselle beverages rich in polyphenols and PCs reported significant increases in the serum levels of SOD and GSH-Px and reduced GSH-Rd, following the daily intake of 200 mL of the beverages (containing 3.92 g of gallic acid and 3.3 g anthocyanins of cyanidin-3-glucoside) for six months. Concurrently, there was also a significant increase in facial skin moisture content among the participants. These findings indicate that roselle beverages contribute to increased antioxidant capacity and improve the skin condition of the participants (65).

In conclusion, the aforementioned research indicates that PCs can mitigate oxidative stress associated with aging skin by neutralizing ROS accumulation, suppressing lipid peroxidation caused by ROS, and enhancing overall antioxidant capacity, as shown in Table 2.

**Table 2:** Antioxidant stress repair effects of PCs on aging skin in different models.

Model	Induction/irradiation dose	PCs intervention	Effects	Mechanism of action	Ref.
Human skin fibroblasts (HSFs) treated with H₂O₂	300 μM H₂O₂, 24 h	25, 50, 100 μg/mL sea buckthorn PCs, 24 h	Cell viability has been significantly enhanced.	PCs significantly enhanced the activities of SOD and GSH antioxidant enzymes, and significantly reduced the levels of ROS and the content of MDA.	(56)
NIH/3 T3 cells treated with etoposide	0.3 μM etoposide, 48 h	1, 3, 10, 30 μM peanut skin PCs, 18 h	The activity of antioxidant enzymes is enhanced, lipid peroxides are reduced, and the number of senescent cells is decreased.	PCs significantly enhanced the activity of SOD antioxidant enzymes, significantly reduced the content of MDA, and thereby significantly lowered the level of ROS.	(57)
Skin of 4-to 6-week-old Kunming mice irradiated with UVB in half male and half female	2.5, 5.0, 7.5 kJ·m^−2^·d^−1^ gradient irradiation, 7 h a day, for 8 weeks	red millet PCs and basal feed were fed in a 1:1 mass ratio, 8 weeks	The activity of antioxidant enzymes was significantly enhanced, and the markers of oxidative stress were significantly reduced.	PCs significantly enhanced the activities of SOD and CAT, thereby significantly reducing the level of ROS by more than 73.3%.	(59)
Skin of 6-to 7-week-old female SKH-1 hairless mice irradiated with UVB	120 mJ/cm^2^, 3 times a week, for 1 month	AIN76A diet supplemented with 0.2 and 0.5% (*w/w*) grape seed PCs was added 2 weeks before UVB irradiation, and continued to be added until the end of the experiment	The activity of antioxidant enzymes was significantly enhanced, and the markers of oxidative stress were significantly reduced.	PCs significantly enhanced the enzymatic activities of GSH, GPx, CAT, significantly reduced the contents of H₂O₂ and MDA, thereby significantly lowering the level of ROS by 40 to 65%.	(60)
Skin of female BALB/C mice (average weight of 20 ± 1 g) irradiated with UVB	240 mJ/cm^2^ once a day for 5 days	5 mg/cm^2^ persimmon PCs, apply locally, 30 min before each UV-B irradiation, 5 h	The symptoms of skin damage such as erythema, wrinkles and thickening of the epidermis on the dorsal skin of the mice were significantly alleviated.	PCs significantly increased the GSH content by 49.2 mM/mg.pro, significantly increased the SOD activity by 39.7 U/mg.pro, significantly restored the GSH-PX activity by 21.7%, significantly reduced the MDA level by 68.9%, and ultimately significantly reduced the accumulation of ROS.	(64)
39 healthy adults aged 40 to 75	Simulate the naturally aging of the skin	Took 200 mL of rose flower beverage (containing 3.92 g of gallic acid, 3.3 g of cyanidin-3-glucoside) orally every day for 6 months	The facial moisture and microcirculation of the subjects’ skin were significantly improved.	The total antioxidant capacity (TEAC) of serum in the extract group was significantly increased by 1.08%, the activities of antioxidant enzymes GSH, SOD and GSH-PX were significantly increased by 40.7, 14.9 and 17.4%, respectively, and the level of thiobarbituric acid reactive substances (TBAR) was significantly decreased by approximately 21.6%. Ultimately, the skin moisture was significantly enhanced by approximately 18.9%.	(65)

### 3.2 PCs increase the content of collagen and elastin in aging skin

ROS generated by skin aging can facilitate the degradation of collagen and elastin through the activation of the mitogen-activated protein kinase (MAPK) signaling pathway, thereby contributing to the process of skin aging. ROS activate receptor tyrosine kinases (RTKs) via oxidative modification, which in turn leads to the activation of MAPKs (66). The MAPK family comprises extracellular signal-regulated kinase (ERK), p38, and c-Jun N-terminal kinase (JNK). ERK promotes the expression of the oncogenic factor c-Fos, whereas both p38 and JNK are involved in activating the transcription factor c-Jun (67, 68). C-Jun dimerizes with c-Fos to form activator protein-1 (AP-1) (69). AP-1 subsequently enhances the upregulation of MMPs such as MMP-1, MMP-3, and MMP-8 (70). The expression of matrix metalloproteinases (MMPs) can be inhibited by tissue inhibitors of metalloproteinase-1 (TIMP-1) but activated by epidermal growth factor receptors (EGFRs) and cytokine receptors. These enzymes play crucial roles in degrading collagen within the extracellular matrix, ultimately leading to structural damage in the skin (71–73).

PCs inhibit the MAPK signaling pathway, thereby suppressing the expression of MMPs and increasing the levels of tissue inhibitors of TIMP-1. This mechanism ultimately prevents collagen and elastin degradation in the skin, promoting the repair of aging skin. Liu et al. (74) investigated the ability of sea buckthorn PCs to repair D-galactose-induced skin aging in mice and reported that they slowed collagen and elastin degradation by increasing TIMP-1 levels in skin tissue while simultaneously inhibiting MMP-1, MMP-3, and MMP-9 expression, thereby improving the conditions associated with skin aging. Michael et al. (75) examined the effects of various concentrations of low-bush blueberry proanthocyanidin enrichment on DU145 human prostate cancer cells. The results demonstrated that this enrichment induced TIMP-1 activity in a dose-dependent manner, leading to reduced MMP expression.

Additionally, findings revealed that grape seed extract inhibited the phosphorylation of proteins such as ERK, JNK, and P38 in human epidermal keratinocytes subjected to UVB irradiation, suggesting its ability to suppress the MAPK signaling pathway and consequently reduce UVB-induced collagen degradation (60, 76). Weng et al. (77) reported a decrease in the gene expression of *MMP-2* and *MMP-9* alongside an increase in TIMP1 expression in murine skin exposed to UVB radiation when 20 mg/kg areca nut PCs were orally administered to mice, indicating their ability to mitigate collagen degradation and slow photoaging effects on the skin through the inhibition of the MAPK signaling pathway. Shi et al. (64) investigated the effects of persimmon PCs on the photoaging of mouse skin exposed to UVB radiation. Research has shown that external application of 5 mg/cm^2^ can reduce the gene expression of MMP-1 and MMP-8, indicating that persimmon PCs may alleviate the expression of the MMP by inhibiting the MAPK signaling pathway, thereby potentially reducing collagen degradation and slowing the photoaging process in the skin. Moreover, UV radiation and ROS can inhibit collagen synthesis by suppressing the TGF-*β*/Smad signaling pathway (78). In fibroblasts, TGF-β1 binds to its specific receptor complexes—TβR I (the receptor for TGF-β type I) and TβR II (the receptor for TGF-β type II). Activation occurs when TβR II phosphorylates TβR I, which subsequently rephosphorylates Smad2/3. Phosphorylated Smad2/3 forms complexes with Smad4, a transcription factor that regulates type I collagen synthesis. Upon entering the nucleus, Smad4 promotes collagen synthesis through transcriptional regulation (79, 80). However, exposure to excessive UVA irradiation results in the upregulation of Smad7 in fibroblasts. This protein subsequently interacts with T*β*R I to prevent the activation of Smad2/3, thereby inhibiting collagen synthesis (81).

PCs can also promote collagen synthesis by activating the TGF-β/Smad signaling pathway, thereby mitigating skin aging. Liu et al. (56) established a natural aging model by treating human skin fibroblasts with varying concentrations of H₂O₂. Following treatment with sea buckthorn PCs, they observed an increase in collagen synthesis through the activation of the TGF-β1/Smad pathway, resulting in notable antiaging effects on the skin. In experiments involving a mouse model of skin aging induced by D-galactose, treatment with higher concentrations of sea buckthorn PCs (50 and 100 mg/kg) led to an increase in TGF-β1 levels within the skin tissue and the upregulation of Smad3 expression. This process promotes collagen and elastin synthesis, augments the content of collagen fibers and elastic fibers in the dermis, and improves signs associated with skin aging (74). Sang et al. (82) investigated the protective effects of PCs extracted from black soybean seed coats on skin fibroblasts and reported that this extract significantly reduced UV radiation-induced apoptosis and intracellular ROS generation while decreasing Smad3 mRNA expression. Additionally, it increased Smad7 mRNA expression to inhibit UV-induced collagen degradation, confirming that PCs enhance skin conditions via activation of the TGF-*β*/Smad signaling pathway, thus indicating its potential for delaying skin aging. Lu et al. (83) treated human skin fibroblasts exposed to UVB radiation with blackcurrant PCs and reported that this treatment elevated TGF-*β* expression in cells post-UVB exposure. Concurrently, it facilitated the phosphorylation of Smad2/3 downstream from TGF-β, enhancing transduction within the TGF-β signaling pathway, which subsequently promoted increased collagen content in these cells, thereby achieving significant antiphotoaging effects on the skin.

In conclusion, the findings of the present study indicate that PCs can alleviate the degradation of collagen and elastin by inhibiting the MAPK signaling pathway. Additionally, it promotes collagen synthesis through the activation of the TGF-β/Smad signaling pathway. As a result, this leads to an increase in the collagen and elastin contents in aging skin, thereby slowing the process of skin aging, as shown in Table 3.

**Table 3:** Repair effects of PCs on collagen and elastin in the aging skin of different models.

Model	Induction/irradiation dose	PCs intervention	Effects	Mechanism of action	Ref.
Skin of 8-week-old female Kunming mice induced by D-galactose	Subcutaneous injection of D-galactose (500 mg/kg) daily, for 7 weeks	25, 50, 100 mg/kg sea buckthorn PCs, administered by gavage, 4 weeks	The hyaluronic acid and water content in the skin tissue of mice increased significantly, the breakage of collagen fibers decreased, and the elastic fibers were distributed intact and orderly.	PCs significantly increased the expression levels of TGF-β1 by 60–80% and TIMP-1 by 40–50%, while significantly reducing the contents of MMP-1, MMP-3, and MMP-9 by 40–60% in 100 mg/kg PCs group, thereby significantly reducing the degradation of collagen by 40–50% and elastin by 50–60%.	(74)
Skin of 6-to 7-week-old female SKH-1 hairless mice irradiated with UVB	120 mJ/cm^2^, 3 times a week for 1 month	Diet supplemented with 0.2 and 0.5% (w/w) grape seed PCs was added 2 weeks before UVB irradiation, and continued to be added until the end of the experiment	The degradation of collagen and elastin is significantly reduced.	0.5% PCs significantly reduced the expression of 67.5% MMPs by significantly decreasing ERK1/2 by 1.5 times and p38 phosphorylation by 0.8 times.	(60)
in of 8-week-old CD-1 female mice irradiated with UVB	130 mJ/cm^2^, 3 times a week for 3 weeks	10, 20 mg/kg areca nut PCs, orally administered by gavage, once a day, 10 days before the start of UVB irradiation and continued until 24 h after the last UVB exposure	The epidermal thickness was significantly reduced, the collagen fiber structure was more complete, and the density was significantly increased.	PCs significantly inhibited the MAPK signaling pathway, significantly downregulated the expression of MMP-9 by 60%, significantly reduced collagen degradation, and maintained the structural integrity of type I collagen.	(77)
Human skin fibroblasts (HSFs) treated with H₂O₂	300 μmol/L, 24 h	25, 50, 100 μg/mL sea buckthorn PCs, 24 h	The synthesis of type I collagen and type I procollagen was significantly upregulated.	100 μg/mL PCs significantly increased the expression of TGF-β1 by 2.1 times, promoted the phosphorylation of Smad2/3, significantly enhanced the nuclear transport of Smad4 and p-Smad3, and thereby increasing the expression of type I collagen and type I procollagen by approximately 2.3 times.	(56)
Skin of 8-week-old female Kunming mice induced by D-galactose	Subcutaneous injection of 500 mg/kg D-galactose once a day for 7 weeks	25, 50, 100 mg/kg Sea buckthorn PCs, once daily for 4 weeks	The collagen fibers and elastic fibers are closely arranged and their quantities increase, and the content of type I collagen and tropoelastin is significantly upregulated.	PCs significantly increased the expression levels of TGF-β1 and Smad3 by activating the TGF-β1/Smad3 pathway, thereby promoting the synthesis of 70–90% collagen and 50–60% elastin precursors.	(74)
Human dermal fibroblast cell (NHDF) irradiated with UVB	Total irradiance dose: 144 mJ/cm^2^	1 μg/mL, 10 μg/mL, 100 μg/mL blackcurrant PCs, 1.5 h	The synthesis of type I procollagen was significantly upregulated.	PCs significantly upregulated the expression of TGF-β and promoted the phosphorylation of Smad2/3, thereby promoting the synthesis of type I procollagen (increasing by 273.9% at 10 μg/mL PCs and 340.1% at 100 μg/mL PCs).	(83)

### 3.3 PCs alleviate the inflammatory response in aging skin

The transcription factor nuclear factor-κB (NF-κB) plays a crucial role in regulating a significant number of genes associated with inflammation and immune responses. It exists as a trimer composed of IκB-*α*, P50, and P65. ROS can phosphorylate and activate IκB kinase (IKK), thereby initiating the NF-κB signaling pathway (84). This activation facilitates the synthesis and secretion of various inflammatory factors by epidermal and dermal cells, including interleukin-1 (IL-1), interleukin-6 (IL-6), cyclooxygenase-2, and tumor necrosis factor-alpha (TNF-α), which collectively induce inflammatory responses (85, 86). Additionally, NF-κB serves as a downstream target within the MAPK pathway. The expression of ERK, p38 MAPK, and AP-1 can increase transcriptional activity within the NF-kB pathway, consequently triggering skin inflammation and accelerating the process of skin aging (87).

PCs have been demonstrated to inhibit the NF-kB signaling pathway, thereby suppressing the production of inflammatory factors associated with this pathway and alleviating the inflammatory response observed in aging skin, as shown in Table 4.

**Table 4:** PCs inhibit inflammatory responses in different models.

Model	Induction/irradiation dose	PCs intervention	Effects	Mechanism of action	Ref.
Primary rat dermal fibroblasts (RDF) treated with H₂O₂	0.3 mM H₂O₂, 30 min	5, 10, 25 μg/mL black rice seed coat PCs, 2.5 h	The cellular inflammatory response was significantly alleviated.	25 μg/mL PCs significantly inhibited the phosphorylation of IκBα and blocked the nuclear translocation of NF-κB p50/p65, thereby reducing the IL-6 inflammatory factor by 1.86 times.	(88)
Normal human epidermal keratinocytes (NHEK) irradiated with UVB	30 mJ/cm^2^, after a single instantaneous irradiation, culture for 24 h	10, 20, 30, 40, 50 μg/mL grape seed PCs, among which 30 μg/mL is the main effective concentration, pretreat for 3–6 h before irradiation, and wash off after irradiation	The cellular inflammatory response was significantly alleviated.	30 μg/mL PCs significantly inhibited the activation of IKKα and the degradation of IκBα, significantly reduced the nuclear translocation of NF-κB/p65, thereby blocking the transcription of downstream inflammatory genes and alleviating the inflammatory response.	(76)
Normal human epidermal keratinocytes (NHEK) irradiated with UVB	30 mJ/cm^2^, after a single instantaneous irradiation, culture for 24 h	5, 10 μg/mL Persimmon Oligomeric Proanthocyanidins, 24 h	The inflammatory response of cells was significantly alleviated, and the apoptosis rate of cells decreased.	10 μg/mL Persimmon Oligomeric Proanthocyanidins significantly inhibited the phosphorylation of p65 and prevented its nuclear translocation, thereby downregulating the expression of 50% TNF-α and 40% IL-6, and thus alleviating the inflammatory response.	(64)
Skin of 6-7-week-old female SKH-1 hairless mice irradiated with UVB	180 mJ/cm^2^, 3 times a week for 24 weeks	0.2, 0.5% (w/w) grape seed PCs was added to the AIN 76A feed for 24 weeks	The degree of white blood cell infiltration in the dermis of the skin was significantly reduced. The expression of UVB-induced proinflammatory cytokines in mouse skin and skin tumors was significantly inhibited.	0.5% PCs significantly downregulated the expression of COX-2. The expressions of pro-inflammatory cytokines such as TNF-α, IL-1β, and IL-6 were significantly reduced by 47, 59, and 58%, respectively, blocking the transmission of inflammatory signals and reducing the stimulation of inflammatory mediators on skin cells.	(89)
66 healthy white women aged 35 to 65, with obvious crow’s feet, mild to moderate sagging and uneven skin tone	Simulation of natural aging skin and photoaging skin (without artificial UV irradiation)	The treatment group Took 100 mg of fermented bilberry extract rich in PCs orally every day, for 28, 56 and 84 days, respectively	On the 84th day, the wrinkle depth, the skin roughness, the skin’s redness component on the subjects’ skin decreased by 10.6, 7.3, 16.8%, respectively; the skin smoothness, firmness, elasticity antioxidant capacity increased by 7.9, 13.3, 12.4, 20.8%, respectively	PCs reduced the skin’s redness component. It is speculated that this might be due to their ability to downregulate the expression of pro-inflammatory factors (such as IL-6), thereby alleviating skin inflammatory responses.	(90)

*In vitro* cell experiments have demonstrated that PCs can alleviate the inflammatory response associated with aging skin. Treatment of H₂O₂-stimulated primary rat skin fibroblasts with PCs extracted from rice inhibited the nuclear translocation of the NF-kBp50/p65 heterodimer within these cells. This inhibition results in reduced inflammatory responses while promoting collagen formation and inhibiting collagen degradation at the cellular level, ultimately contributing to a deceleration of the skin aging process (88). Sudheer et al. (76) reported that grape seed PCs could inhibit phosphorylation events involving ERK, JNK, and p38 in human epidermal keratinocytes following UVB irradiation; they also inhibited both the activation effects and the nuclear translocation of NF-kBp50/p65. These findings suggest that grape seed PCs may effectively reduce UVB-induced skin inflammation through the suppression of NF-kB pathway transcription. Xin et al. (64) demonstrated that persimmon oligoproanthocyanidins mitigate skin inflammatory responses by inhibiting the activation of the NF-kB signaling pathway in UVB-damaged human keratinocyte HaCaT cells and reducing the expression of inflammatory cytokines, including TNF-*α* and IL-6.

Additionally, *in vivo* animal experiments and a clinical trial confirmed that PCs can alleviate the inflammatory response associated with aging skin. Sharma et al. (89) administered grape seed PCs of 0.2 and 0.5% (w/w) in the diet to hairless SKH-1 mice exposed to UVB radiation. The experimental findings demonstrated that grape seed PCs inhibited the activation and nuclear translocation of NF-kB while downregulating the expression of proinflammatory cytokines such as TNF-*α*, IL-1, and IL-6, as well as cyclooxygenase-2. These findings suggest that grape seed PCs exert an antiskin aging effect by inhibiting the NF-kB signaling pathway, thereby mitigating inflammatory responses and reducing skin collagen degradation. Similar outcomes were reported in a study conducted by Anshu et al. (58) where SKH-1 hairless mice were irradiated with UVB and subsequently administered grape seed PCs. The results indicated that grape seed PCs significantly reduced the levels of inflammatory factors such as TNF-α and IL-6 within the skin of these mice. In a clinical trial investigating the antiaging, brightening, and antioxidant effects of fermented bilberry extract (FBE) containing PCs, 66 female participants aged 35 to 65 years who exhibited prominent wrinkles along with mild to moderate skin laxity and uneven skin tone were enrolled. The participants were randomly assigned to either the FBE group receiving 100 mg of fermented bilberry extract daily or the placebo group receiving maltodextrin and magnesium stearate devoid of PCs. These findings revealed that FBE enhanced the antioxidant capacity of the skin while concurrently reducing inflammation (90).

### 3.4 PCs inhibit pigmentation in aging skin

UV radiation is the primary inducer of melanin production. Prolonged exposure to UV radiation can lead to an increase in the number of melanocytes within the skin, thereby increasing melanin synthesis and resulting in skin pigmentation (9, 91). Specifically, UVA radiation activates opsin receptors in melanocytes, which subsequently promotes melanin synthesis. Conversely, UVB radiation enhances melanin synthesis by facilitating melanocyte differentiation and stimulating keratinocytes to release melanocyte-stimulating hormone (MSH), along with associated immunomodulatory factors and stem cell growth factors (92, 93). Upon exposure to UV radiation, endogenous *α*-melanocyte stimulating hormone (α-MSH) binds to the melanocortin 1 receptor (MC1R). This interaction activates adenylate cyclase (AC), leading to an increase in intracellular cyclic adenosine monophosphate (cAMP) levels. Subsequently, cAMP activates protein kinase A (PKA), which ultimately mediates the activation of cAMP response element binding protein (CREB) and microphthalmia-associated transcription factor (MITF). These transcription factors increase the expression of Tyrosinase (TYR), a key enzyme involved in melanin synthesis, thereby stimulating melanin production (94, 95).

PCs can inhibit the activity of tyrosinase, leading to a reduction in tyrosine expression and a decrease in melanin synthesis. This process ultimately results in diminished melanin deposition within the body and contributes to the slowing of skin aging. Chong et al. (96) treated thyroxine-induced senescent mice with grape seed PCs and reported that these PCs significantly inhibited tyrosinase activity in the skin cells of these aged mice, thereby suppressing melanin synthesis and effectively alleviating skin pigmentation issues. Matthew et al. (97) demonstrated that tyrosinase activity was markedly reduced when PCs extracted from lychee roots were used, suggesting that these PCs could achieve skin whitening effects by inhibiting tyrosinase activity. Chen et al. (63) explored the inhibitory effect of lotus seed PCs on melanin synthesis in human skin fibroblast epithelial cells exposed to UVB radiation. Their research revealed that lotus seed PCs effectively suppressed the conversion of melanin precursors (such as tyrosine and DOPA) into melanin by inhibiting the activity of tyrosinases, including monophenolase and diphenolase, thus significantly reducing UV-induced pigmentation.

In a clinical trial designed to evaluate the effects of apple extract (AP) supplements containing PCs on skin pigmentation issues induced by UV exposure in healthy women, participants were randomly assigned to three groups: a low-dose AP group (300 mg/day), a high-dose AP group (600 mg/day), and a placebo group. These groups underwent continuous supplementation for 12 weeks. The results indicated that AP administration significantly reduced the melanin content of the skin following UV exposure, confirming its capacity to diminish melanin production through the inhibition of tyrosinase activity (98). In another clinical trial investigating the impact of red wine beverages containing PCs on sunburn and dryness resulting from UV exposure in women, 100 healthy female subjects were randomly allocated into two groups: the test group receiving 200 mg/day of PCs-containing red wine beverages and the control group receiving 200 mg/day of red wine beverages devoid of PCs. After 12 weeks of continuous consumption, measurements revealed a significant reduction in the melanin index at sunspots within the test group, indicating that PCs-containing red wine beverages effectively inhibited tyrosinase activity, leading to decreased melanin production (99).

In addition to the previously mentioned four beneficial effects on aging skin, PCs can also enhance barrier function and hydration. They promote the upregulation of filaggrin and keratin expression. Filaggrin plays a crucial role in lipid synthesis within the stratum corneum of the skin, whereas keratin is essential for the formation of the skin barrier. Consequently, PCs contribute to improved moisture retention in the skin and strengthen its resistance to external stimuli (100, 101). Furthermore, PCs can facilitate cell migration, which is a key aspect of tissue repair. In fibroblasts subjected to oxidative stress from H₂O₂ exposure, treatment with PCs significantly increased cell migration rates. This enhancement enables these cells to resynthesize extracellular matrices such as collagen, thereby directly supporting skin regeneration and repair while delaying collagen imbalance-related skin aging (56, 102).

## 4 Methods for improving the stability and bioavailability of PCs

PCs exhibit poor stability, which significantly diminishes their bioavailability. To address this limitation, encapsulation techniques can be employed to increase the stability of PCs, thereby improving their bioavailability (103, 104). Encapsulation refers to the process of embedding bioactive compounds within solid particles or liquid vesicles. This technique not only regulates the release of bioactive substances but also masks undesirable odors, stabilizes biological activity, and protects sensitive compounds from degradation (105, 106). Currently, encapsulation is a focal point of research with extensive application potential. It is widely acknowledged as an effective strategy for preserving the stability of bioactive substances and extending the shelf-life of materials that are sensitive to light and heat (107, 108). The primary forms of encapsulation for PCs include microcapsules, liposomes, and nanoparticles, as shown in Table 5.

**Table 5:** Packaging types for stabilizing PCs.

Encapsulation type	Materials	Function	Advantage	Ref.
Microcapsule	Sodium alginate Carboxymethyl cellulose	Isolate oxygen, light and damage from gastric acid	Effectively delay the release of PCs, stability and bioavailability of PCs ↑	(132)
	Polylactic acid	Isolate the PCs from direct contact with the external environment and control the release speed of the PCs	Stability and bioavailabilityof PCs ↑	(133)
	Sodium alginate	Isolate the degradation of PCs by light and oxygen	Antioxidant activity, stability and bioavailability of PCs↑	(134)
Liposomes	Maltodextrin β-cyclodextrin	Isolate oxygen, light and damage from gastric acid	Target the release of PCs into the intestine to prevent degradation by gastric acid	(113)
	Soy lecithin Cholesterol	Isolate the PCs from direct contact with the external environment and control the release rate of the PCs	Solubility, stability and bioavailability of PCs↑	(135)
	Soy lecithin Cholesterol	The packaged PCs have better ROS removal activity	Water solubility, stability and bioavailability of PCs↑	(136)
Nanoparticles	Casein-maltodextrin	Slow down the decomposition of PCs in the gastrointestinal tract and enhance its ability to resist oxidative stress and heat shock	Antioxidant stability and bioavailability of PCs↑	(137)
	Lecithin	Improve the packaging efficiency of PCs and stabilize their antioxidant capacity	Control PCs release and stability↑	(138)
	Soy phosphatidylcholine Monoglyceride citrate	Improve the packaging efficiency of PCs	Stability and bioavailability of PCs ↑	(139)
	Low-molecular-weight chitosan	Form stable and highly bioactive nanoparticles	Biological activity of PCs ↑	(140)

Microcapsule technology represents an innovative packaging approach that has emerged in recent years. This technique primarily involves the dispersion of small-molecule active compounds, sensitive materials, or volatile substances through physical or chemical methods, followed by the formation of microcapsules encapsulated with protective films (109). First, these microcapsules effectively shield bioactive components from environmental degradation, prolong their stability, and ensure precise delivery to targeted sites. Additionally, they minimize interactions between different ingredients, thereby extending the shelf-life of the product. Owing to these advantages, microcapsule technology has found extensive applications across diverse fields, such as food science, biomedicine, sensing technologies, and textiles (110–112). Moreover, microencapsulation can enhance the light and oxidation resistance of PCs, thereby broadening their potential and application range in functional products (113).

Liposomes play a crucial role in colloidal drug delivery systems, particularly within the fields of food and nutrition. Primarily composed of phospholipids, they can encapsulate water-soluble substances in their internal aqueous phase while accommodating lipophilic compounds within the lipid bilayer, thereby forming liposomes with a well-defined bilayer structure (114, 115). These structures exhibit several advantageous properties, including low toxicity, high plasticity, complete biodegradability, lack of immunogenicity, self-assembly capability, and ease of modification (115). Given that PCs are water soluble, they can be effectively incorporated into the aqueous core of liposomes. This incorporation protects PCs from degradation caused by environmental factors such as light exposure, pH fluctuations, and temperature variations (113).

Nanoparticles have been shown to be an optimal choice for encapsulating phenolic compounds and enhancing their bioavailability. They significantly improve the uptake efficiency of polyphenols by increasing their solubility, facilitating their absorption through endocytosis, and promoting the entrapment or surface adsorption of biomolecules (116). This encapsulation technology not only enhances the solubility and stability of bioactive compounds but also plays a pivotal role in drug delivery systems. The carrier materials used for nanoparticles are typically biodegradable or ion sensitive and exhibit low toxicity, minimal side effects, and controllable release characteristics. Moreover, nanoparticles can traverse intercellular and intertissue gaps to reach target sites directly, thereby further improving the bioavailability of bioactive substances (117–119).

In addition, the intestinal microbiota, dietary fiber and fat, various antioxidants, pH regulation, and low-temperature extraction methods can increase the bioavailability of PCs. PCs operate synergistically with intestinal microorganisms such as *Lactobacillus* and *Bifidobacterium*. The metabolism of PCs by these intestinal microorganisms results in the generation of short-chain fatty acids and other secondary metabolites that further augment the antioxidant effects of PCs (120). Moreover, PCs exhibit a synergistic relationship with dietary fiber and fat. When combined with dietary fiber, PCs are protected from degradation by digestive enzymes during digestion; this facilitates their gradual release in the intestines, thereby prolonging their antioxidant activity (121). Additionally, when PCs are paired with other antioxidants, such as vitamin C, they promote preferential oxidation of these antioxidants, which enhances the bioavailability of PCs (122). The stability and bioavailability of PCs in aqueous solutions can be improved by incorporating an acidic buffer to adjust the pH to acidic levels or by avoiding mixing the PCs solution with alkaline substances (46). Furthermore, employing low-temperature extraction techniques for PCs—such as low-temperature prefmentation impregnation and dry ice freezing—can minimize degradation during extraction processes. These approaches collectively improve both the stability and bioavailability of polyphenol compounds (123).

## 5 Summary and outlook

**Figure 3:**
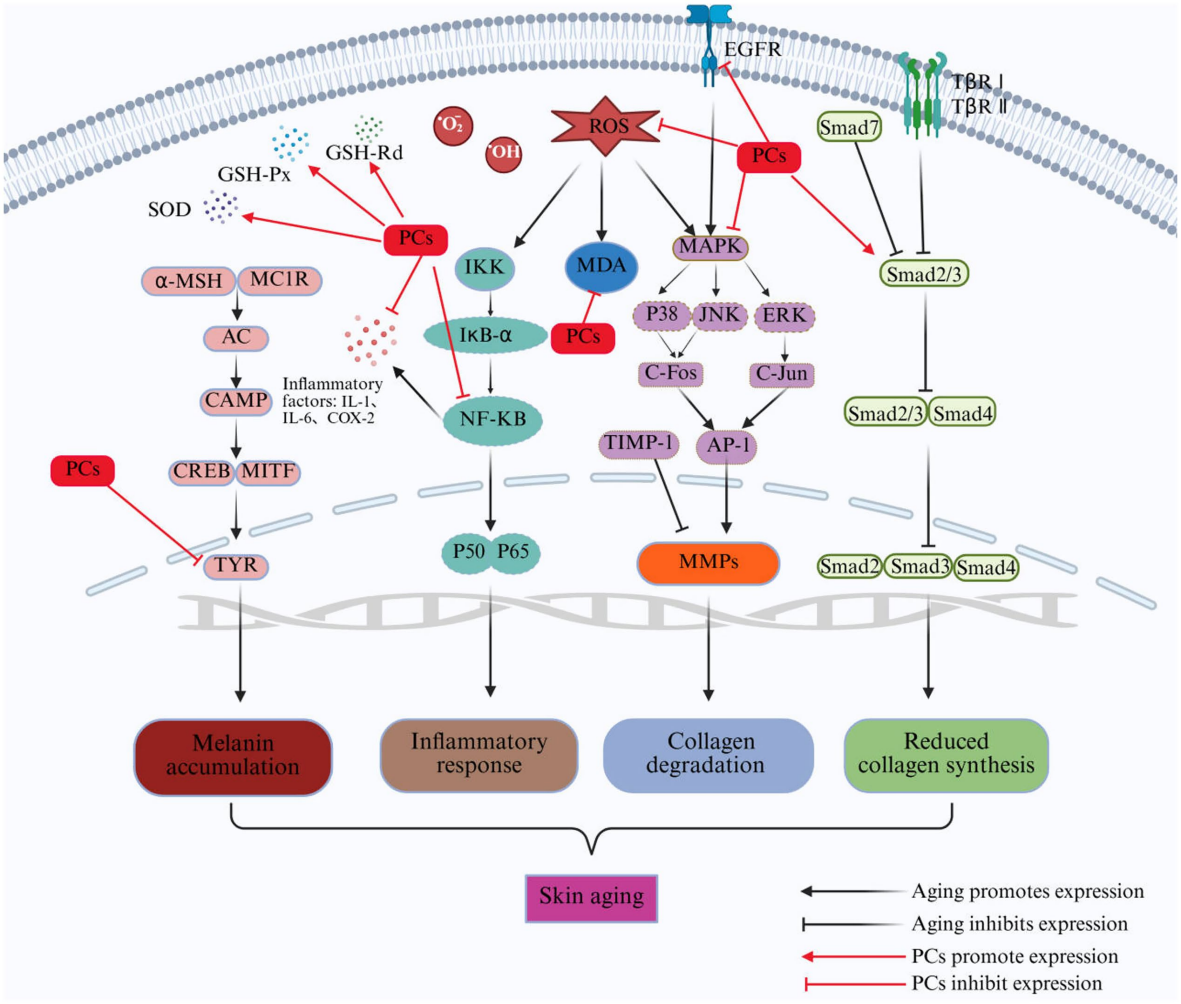
Repair mechanism of PCs in aging skin. ROS, reactive oxygen species; O_2_^−^, superoxide radical; OḤ, hydroxyl radical; MAPK, mitogen-activated protein kinase; ERK, extracellular signal-regulated kinase; JNK, C-Jun N-terminal kinase; AP-1, activator protein-1; IKK, inhibitor of Kappab kinase; I*k*B-*α*, NF-kappa-B Inhibitor Alpha; TβR I, transforming growth factor-β receptor I; TβR II, transforming growth factor-β receptor II; Smad, small mothers against decapentaplegic proteins; MMPs, matrix metalloproteinases; α-MSH, α-melanocyte stimulating hormone; MC1R, melanocortin 1 receptor; AC, activate adenylate cyclase; cAMP, cyclic adenosine monophosphate; TYR, tyrosinase; MDA, malondialdehyde; TIMP-1, tissue inhibitor of metalloproteinases 1; SOD, superoxide dismutase; GSH-Px, glutathione peroxidase; GSH-Rd, glutathione reductase.

Skin aging is a multifaceted biological process influenced by various factors. Currently recognized contributors to this phenomenon include increased oxidative stress, elevated expression of MMPs, degradation of collagen and elastin, heightened inflammatory responses, and melanin deposition, among others. The mechanisms through which PCs exert anti-skin aging effects involve multiple targets, levels, and pathways, as shown in Figure 3. PCs can alleviate oxidative stress in aging skin by neutralizing ROS, inhibiting ROS-induced lipid peroxidation, and enhancing the antioxidant capacity of the skin. Furthermore, it reduces the degradation of collagen and elastin in aged skin while promoting collagen synthesis by inhibiting the MAPK pathway and activating the TGF-*β*/Smad signaling pathway. This dual action results in increased levels of collagen and elastin within aging skin. Additionally, PCs mitigate the inflammatory response associated with skin aging by inhibiting the NF-kB signaling pathway, thereby decreasing the secretion of proinflammatory cytokines. Moreover, it inhibits tyrosinase activity to reduce melanin production in aged skin, effectively suppressing pigmentation.

With increasing awareness of public health and ongoing in-depth research on PCs, the development and application of PCs present significant opportunities. PCs not only delay and repair skin aging but also exhibit remarkable efficacy in combating skin cancer, alleviating allergic reactions on the skin, protecting cardiovascular health and cerebrovascular functions, preventing myopia development, and relieving physical fatigue and mental distress, such as depression. However, it is important to acknowledge that there are certain limitations and knowledge gaps in the current research regarding the anti-skin aging effects of PCs. The limitations of existing research are reflected in several key aspects: (1) Lack of clinical research: Most studies investigating the antiaging effects of PCs rely on *in vitro* cell culture or *in vivo* animal models. While these models provide valuable preliminary data, they may not fully capture the complexity of human skin physiology and metabolism. Additionally, clinical trials often involve relatively small sample sizes, which limits both the statistical power and generalizability of their findings. (2) Verification is needed for nanoparticles validity: PCs exhibit poor stability under various environmental conditions, such as high temperature, light exposure, and alkalinity. This instability significantly impacts their bioavailability and efficacy. Although packaging techniques have been explored to increase stability, their effectiveness in improving the bioavailability of PCs within the human body remains inadequately understood. (3) Unresolved mechanism underlying photoaged skin repair: Although some mechanisms by which PCs combat skin aging have been partially elucidated, a comprehensive understanding of their molecular and cellular interactions is still lacking. In particular, further detailed investigations into the signaling pathways and gene networks regulated by PCs within human skin are necessary for advancing this field.
